# Spatial heterogeneity in liver stiffness does not predict clinical outcomes in patients with primary sclerosing cholangitis

**DOI:** 10.1097/HC9.0000000000000821

**Published:** 2025-10-07

**Authors:** Erick Cruz Grave, Connie Chan, Michael T. Corwin, Richard Dean, Sandeep Dhaliwal, Maryam Yazdanfar, Christopher L. Bowlus

**Affiliations:** 1 Division of Gastroenterology and Hepatology, University of California Davis School of Medicine, Sacramento, California, USA; 2 Department of Internal Medicine, University of Southern California, Los Angeles, California, USA; 3 Department of Radiology, University of California Davis School of Medicine, Sacramento, California, USA

**Keywords:** imaging, magnetic resonance elastography, predictors, survival

## Abstract

**Background:**

Primary sclerosing cholangitis (PSC) is a cholestatic liver disease that can cause uneven fibrosis throughout the liver. Spatial heterogeneity in liver stiffness (LS) by magnetic resonance elastography (MRE) was compared in patients with PSC and metabolic dysfunction–associated steatotic liver disease (MASLD).

**Methods:**

Variability of LS was defined as the difference between the maximum and minimum LS divided by the maximum LS. The coefficient of variation (CoV) was calculated as the SD of LS divided by the mean of LS. MREs were classified as homogenous or heterogenous if the variability of LS was less than or greater than the mean variability, respectively.

**Results:**

A total of 105 patients (PSC: n=66, MASLD: n=39) were included. In both PSC and MASLD, the variability of LS increased with increasing mean LS (*r=*0.31, *p*=0.01 and *r*=0.57, *p*=0.0002, respectively). CoV correlated with mean LS in patients with MASLD (*r*=0.34, *p*=0.03), but not PSC (*r*=0.19, *p*=0.12). Among patients with PSC, neither variability nor CoV of LS were predictors of transplant-free survival (TFS) or hepatic decompensation (HD). Mean LS was a significant predictor of TFS (HR 1.52, *p*=0.004) and HD (HR 1.94, *p*<0.0001), independent of LS variability or CoV.

**Conclusions:**

Spatial heterogeneity of LS increases with progressive disease but is not associated with clinical outcomes in PSC. Mean LS predicts clinical outcomes in patients with PSC independent of LS spatial heterogeneity.

## INTRODUCTION

Primary sclerosing cholangitis (PSC) is a chronic cholestatic liver disease with a natural history of disease progression related to biliary fibrosis and cholestasis resulting in cirrhosis and its complications.^1^ Patients with PSC are also at risk of developing cholangiocarcinoma, gallbladder cancer, bacterial cholangitis, and, in those with concomitant inflammatory bowel disease (IBD), colon cancer. The median transplant-free survival is estimated to be ~20 years.^2^ Current biomarkers of cholestasis and/or liver fibrosis do not fully reflect disease stage or progression and are not adequate for use as surrogate endpoints in clinical trials.^3^

Liver fibrosis estimated directly by histology^4^ or indirectly by liver stiffness (LS) measured by vibration-controlled transient elastography (VCTE)^5^^,^^6^ has been associated with clinical outcomes. However, compared with other liver diseases, fibrosis in PSC has been observed to be heterogenous due to segmental biliary strictures. As a result, even biopsy has proven to be a challenging marker of clinical outcomes.^7^ Unlike a needle biopsy or VCTE, which only samples a small fraction of the liver, magnetic resonance elastography (MRE) measures liver stiffness across the entire liver. Prior studies have demonstrated that LS by MRE is predictive of liver fibrosis and cirrhosis, and changes in LS by MRE are associated with clinical outcomes in patients with PSC.^8^^,^^9^ Prior studies have suggested that LS heterogeneity is greater in PSC compared with viral hepatitis^10^ and in patients with metabolic dysfunction–associated liver disease (MASLD), spatial heterogeneity on MRE results in greater discordance between histologic and LS staging of fibrosis.^11^ Herein, we describe the results of a study aimed at comparing the spatial heterogeneity of LS by MRE between patients with PSC or MASLD and determining the effects of spatial heterogeneity on clinical outcomes in patients with PSC.

## METHODS

This was a single-center retrospective cohort study of patients with PSC or MASLD who had at least 1 MRI study with MRE performed between January 2002 and December 2023. Inclusion criteria for the PSC cohort included a diagnosis of large duct PSC, small duct PSC, or PSC with autoimmune hepatitis features based upon current guidelines.^1^ Inclusion criteria for the MASLD cohort included a diagnosis of MASLD based upon the presence of hepatic steatosis by imaging or biopsy and the absence of significant alcohol use or other cause of hepatic steatosis. Exclusion criteria included secondary sclerosing cholangitis, chronic viral hepatitis or other chronic liver disease, nondiagnostic or absence of MRE imaging. Demographic data, including age, sex, and relevant medical history, including diabetes mellitus, hypertension, or dyslipidemia, were collected from medical records. Laboratory data available within 6 months of index MRE were used to assess time-to-failure analysis of clinical outcomes from hepatic events. A waiver of informed consent was granted by the Institutional Review Board of the University of California, Davis. The study was approved by the local Institutional Review Board and conducted in accordance with the Declarations of Helsinki and Istanbul.

### Magnetic resonance elastography

All patients were instructed to fast for 4 hours before the MRI examination. Images were acquired on a 1.5 Tesla General Electric Signa MRI Scanner system (GE Medical Systems). The MRCP (magnetic resonance cholangiopancreatography) protocol included coronal and transverse single-shot fast spin echo (SSFSE) sequences, a 3D fast spin echo coronal T2-weighted MRCP sequence, and a 2D thick slab T2-weighted MRCP sequence. For cases with IV contrast, transverse pre-phasic and multi-phasic (arterial, portal venous, and delayed) post-contrast T1-weighted 3D spoiled gradient echo pulse (liver acquisition with volume acceleration—LAVA) sequences were acquired. For MRE, a 2-D gradient echo pulse sequence was performed with post-processing to yield quantitative images of tissue shear stiffness in kilopascals. In cases where more than one magnetic resonance elastogram was available, the earliest magnetic resonance elastogram from the time of diagnosis of PSC was included. On each section of the magnetic resonance elastogram from the MRE acquisition, regions of interest (ROIs) were drawn to include parenchyma of the right lobe, avoiding edges of the liver, “hot spots” often seen under the driver, large blood vessels, and areas of poor signal-to-noise ratio as indicated on the elastogram + mask images.

Based on previous literature, 2 methods of heterogeneity scoring were used.^10^^,^^11^ Variability in LS on MRE was defined as the difference between the maximum and minimum 1-cm^2^ ROI of LS divided by the maximum value and expressed as a percentage.^11^ Cases were categorized as homogenous or heterogenous based on whether the variability was less or greater than the mean variability for the entire cohort. The coefficient of variance (CoV) was calculated by taking the average of the ratios of the intrahepatic standard deviations to the corresponding means of LS for each MRE slice.^10^ Fibrosis stage was determined by mean LS using optimal cutoff values of ≤3.4 kPa for F0-1, 3.5–3.9 kPa for F2, 4.0–4.9 kPa for F3, and >5.0 kPa for F4 as previously published and classified as early fibrosis (<4.0 kPa) or advanced fibrosis (≥4.0 kPa).^12^

### Statistical analysis

Descriptive statistics of demographic, clinical, and laboratory variables of the cohort were calculated. Continuous variables were compared using the Mann–Whitney *U* test or Kruskal–Wallis *H* test, and categorical values were compared using the chi-square test. Spearman correlation was used to assess the correlation between LS and heterogeneity values.

Because timing of liver transplantation is dependent on organ allocation which has changed over time, source of organ donation (living vs. deceased), and exceptions for bacterial cholangitis and cholangiocarcinoma, we examined both transplant-free survival (TFS) and the composite outcome of hepatic decompensation (HD) which was defined as the first of any event of ascites, hepatic encephalopathy, variceal bleeding, liver transplantation, and death. Cox proportional hazard regression was used to identify significant variables associated with hepatic decompensation from the time of the first available MRI/MRCP. Kaplan–Meier survival analysis was used to visualize differences in survival. All statistical analyses were performed using Stata version 17.0 (StataCorp). A significance level of *p*<0.05 was set for all analyses.

## RESULTS

### Patient characteristics

The study included 105 patients, of whom 66 had PSC and 39 had MASLD (Table 1). PSC patients had a median age of 45 years (IQR 25), 47 (71%) were male, and IBD was present in 47 (71%). MASLD participants were older, with a median age of 61.0 years (IQR 19) and predominantly female (69%). Patients with PSC had higher ALT and platelets, and lower albumin compared with patients with MASLD. The median (IQR) LS was greater in patients with MASLD compared with PSC [4.2 (3.2) vs. 3.6 (1.7) kPa, respectively, *p*=0.004]. Among the PSC study cohort, 17 patients experienced a first clinical event of hepatic decompensation, including ascites in 8 patients and variceal hemorrhage in 3 patients or liver transplantation, including 3 via deceased donor and 3 via living donor.

**TABLE 1:** Clinical and biochemical characteristics of patients with primary sclerosing cholangitis or metabolic dysfunction–associated steatotic liver disease

	PSC (n=66)	MASLD (n=39)	*p*
Age (y)	45.0 (27.0–52.0)	61.0 (49.0–68.0)	<0.0001
Sex, male	47 (71%)	12 (31%)	<0.0001
Race			0.12
White	34 (52%)	27 (69%)	
Black or African American	4 (6%)	—	
Other	8 (12%)	2 (5%)	
Unknown	20 (30%)	10 (26%)	
PSC type			—
Large duct	64 (96%)	—	
PSC/AIH	1 (2%)	—	
Small duct	1 (2%)	—	
Inflammatory bowel disease			—
Ulcerative colitis	37 (56%)	—	
Crohn disease	10 (15%)	—	
No IBD	19 (29%)	—	
Laboratories			
Sodium (mg/dL)	139.0 (137.0–140.0)	138.5 (137.0–140.0)	0.91
Creatinine (mg/dL)	0.9 (0.7–1.0)	0.8 (0.7–1.0)	0.17
ALP (IU/L)	166.0 (99.5–302.0)	73.0 (65.0–89.0)	<0.0001
ALT (IU/L)	52.0 (35.5–107.0)	38.0 (31.0–61.0)	0.04
AST (IU/L)	44.0 (32.5–79.5)	36.0 (28.0–53.0)	0.05
Total bilirubin (mg/dL)	0.8 (0.5–1.2)	0.8 (0.5–1.0)	0.28
Albumin (g/dL)	3.8 (3.5–4.1)	4.1 (3.7–4.3)	0.02
Platelets (×10^3^/μL)	251.0 (189.0–335.0)	205.0 (123.0–238.0)	0.003
Magnetic resonance elastography			
Liver stiffness (kPa)	3.6 (2.8–4.5)	4.2 (3.0–6.2)	0.04
Advanced fibrosis (F3–F4)	27 (41%)	23 (59%)	0.03
Variability, %	51.0 (43.4–59.9)	46.2 (41.1–60.2)	0.31
Coefficient of variation	21.8 (18.2–27.2)	20.8 (17.0–25.3)	0.52

*Note*: Quantitative variables are expressed as median (IQR). Categorical variables are expressed as an absolute number (percentage).

Abbreviations: AIH, autoimmune hepatitis; IBD, inflammatory bowel disease; MASLD, metabolic dysfunction–associated steatotic liver disease; PSC, primary sclerosing cholangitis.

### Spatial heterogeneity of liver stiffness in PSC and MASLD

**FIGURE 1:**
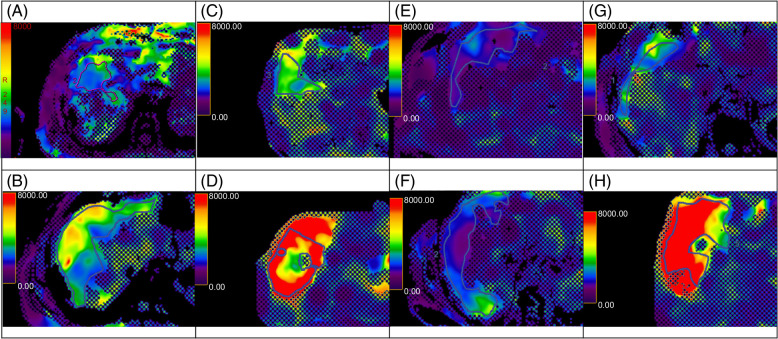
Representative magnetic resonance elastogram slices from patients with primary sclerosing cholangitis (PSC) and metabolic dysfunction–associated steatotic liver disease (MASLD). The top row represents patients with homogeneous spatial variation, and the bottom row patients with heterogeneous spatial variation. Panels A–D are from patients with PSC with low liver stiffness (A and B; 3.14 and 2.20 kPa, respectively) and high liver stiffness (C and D; 4.42 and 8.80 kPa, respectively). Panels E–H are from patients with MASLD with low liver stiffness (E and F; 1.80 and 2.14 kPa, respectively) and high liver stiffness (G and H; 3.3 and 10.1 kPa, respectively).

**FIGURE 2:**
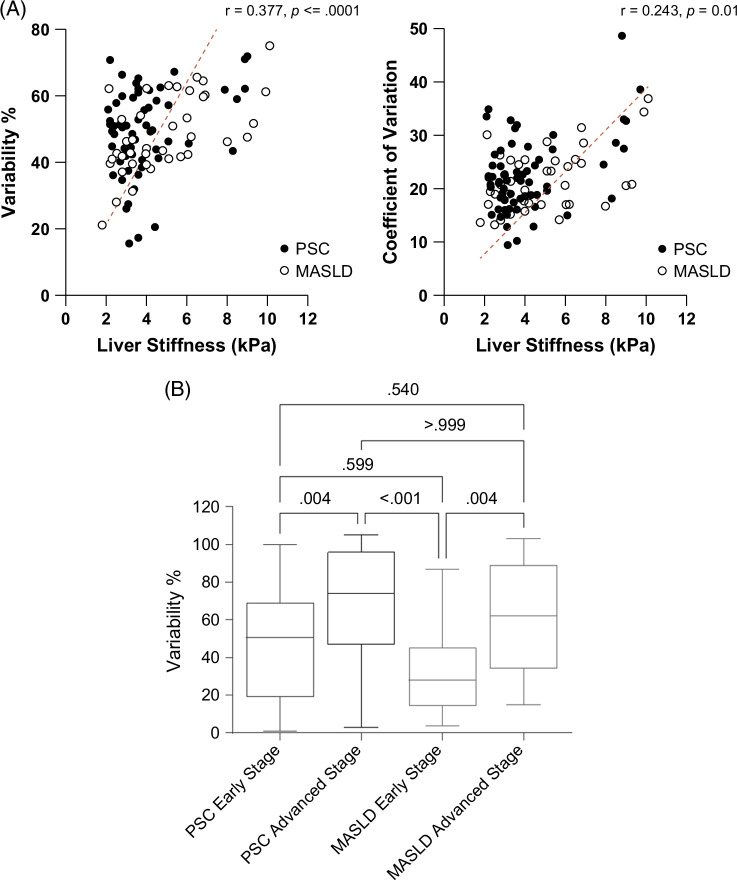
(A) Correlation of mean liver stiffness with spatial heterogeneity measured by variability and coefficient of variance (CoV). (B) Comparison of variability by disease type and early versus advanced-stage disease.

Neither measure of spatial heterogeneity (variability or CoV) differed between patients with PSC or MASLD. (Table 1) In addition, there was no difference in the frequency of heterogenous studies in patients with PSC [35 (53%)] compared with MASLD [16 (41%)] (*p*=0.32). In both PSC and MASLD, spatial heterogeneity increased with increasing LS (Figures 1, 2A).

To determine if spatial heterogeneity differs by stage (LS <4.0 kPa) in PSC compared with MASLD, heterogeneity was compared between patients with early and advanced stages. Patients with advanced PSC showed higher liver stiffness variability [median 57.9% (IQR 17.1)] compared with early-stage PSC [median 48.5% (IQR 15.4), *z*=2.8, *p*=0.004] (Figure 2B). Subjects with advanced MASLD also showed increased variability [median 51.7% (IQR 18.6)] compared with early-stage MASLD [median 42.2% (IQR 8.2), *z*=−2.9, *p*=0.004]. There was no statistically significant difference in CoV between early and advanced stages for PSC (*p*=0.11) and MASLD (*p*=0.17).

### Associations of spatial heterogeneity and LS with biomarkers and prognostic scores

To determine the potential impact of spatial heterogeneity on the ability of MRE to reflect the disease state of PSC, we sought associations between spatial heterogeneity and biomarkers of disease and PSC prognostic models for transplant-free survival (Mayo Risk Score) and hepatic decompensation-free survival (PREsTo score). Among all patients, LS variability was significantly associated with ALP (*r*=0.204, *p*=0.04) and INR (*r*=0.284, *p*=0.02). For CoV, a significant relationship was found only for INR (*r*=0.246, *p*=0.04). LS was associated with total bilirubin (*r*=0.389, *p*<0.0001), ALP (*r*=0.208, *p*=0.04), AST (*r*=0.406, *p*<0.0001), INR (*r*=0.339, *p*=0.005), albumin (*r*=−0.350, *p*=0.0004), and platelet count (*r*=−0.382, *p*=0.0001). (Table 2).

**TABLE 2:** Correlation of liver stiffness mean, variability, and coefficient of variation with laboratory values and prognostic scores

	All patients (n=105)						PSC only (n=66)					
	Mean		Variability		CoV		Mean		Variability		CoV	
	*r*	*p*	*r*	*p*	*r*	*p*	*r*	*p*	*r*	*p*	*r*	*p*
Total bilirubin	*0.389*	*<0.0001*	0.176	0.08	0.097	0.34	*0.408*	*0.001*	0.133	0.29	0.06	0.62
ALP	*0.208*	*0.04*	*0.204*	*0.04*	0.033	0.75	*0.518*	<*0.0001*	0.206	0.10	−0.021	0.87
AST	*0.406*	*<0.0001*	0.197	0.05	0.065	0.52	*0.455*	*0.0002*	0.161	0.21	−0.007	0.96
ALT	0.134	0.19	0.075	0.46	−0.041	0.69	0.214	0.09	0.112	0.38	−0.078	0.54
Albumin	*−0.350*	*0.0004*	−0.202	0.05	−0.068	0.51	*−0.440*	*0.0003*	−0.119	0.35	0.007	0.96
Creatinine	−0.114	0.27	−0.020	0.84	0.113	0.27	0.063	0.63	0.07	0.57	0.200	0.12
INR	*0.339*	*0.005*	*0.284*	*0.02*	*0.246*	*0.04*	0.261	0.08	*0.340*	*0.02*	0.296	0.05
Sodium	−0.126	0.22	−0.014	0.89	0.074	0.47	−0.093	0.47	0.153	0.24	*0.294*	*0.02*
Platelet	*−0.382*	*0.0001*	−0.097	0.35	−0.069	0.51	−0.260	0.06	−0.113	0.39	−0.103	0.43
Mayo risk score	—	—	—	—	—	—	*0.454*	*0.002*	0.218	0.16	0.030	0.85
PREsTo^a^	—	—	—	—	—	—	*0.423*	*0.001*	0.010	0.94	−0.101	0.43

a PREsTo score at 5 years.

Abbreviations: CoV, coefficient of variation; INR, international normalized ratio; PSC, primary sclerosing cholangitis.

Among PSC patients, LS variability was only associated with INR (*r*=0.340, *p*=0.02), and CoV was only associated with sodium (*r*=0.294, *p*=0.02). In contrast, LS was associated with total bilirubin (*r*=0.408, *p*=0.001), ALP (*r*=0.518, *p*<0.0001), AST (*r*=0.455, *p*=0.0002), and albumin (*r*=−0.440, *p*=0.0003). Among PSC prognostic models, LS correlated with Mayo risk score (*r*=0.454, *p*=0.002) and PREsTo score (*r*=0.423, *p*=0.001), but no associations were found with LS variability or CoV with either prognostic score.

### Associations of spatial heterogeneity and LS with biomarkers and clinical outcomes

**FIGURE 3:**
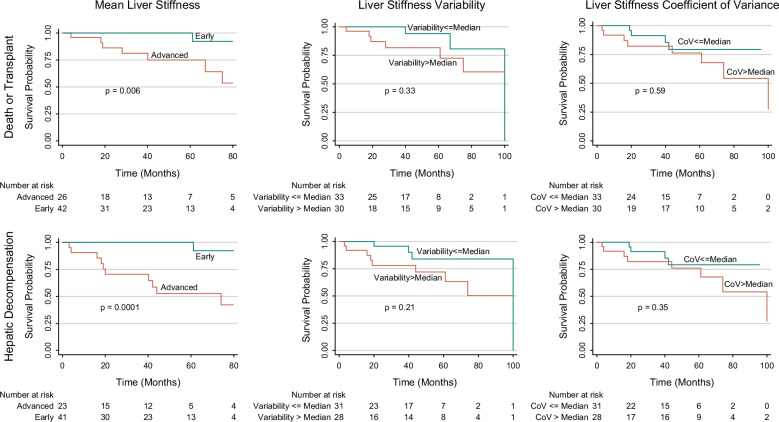
Kaplan–Meier survival curves free of liver transplant (top row) or hepatic decompensation (bottom row) for patients with early versus advanced fibrosis based upon mean liver stiffness defined as a mean < or ≥4 kPa (left column), spatial heterogeneity by variability (middle column), or coefficient of variance (right column).

Patients with PSC who had advanced fibrosis based upon LS had lower transplant-free survival and hepatic decompensation-free survival compared with those with early-stage fibrosis (*p*=0.0001) (Figure 3). The 20-month, 40-month, 60-month, 80-month, and 100-month hepatic decompensation-free survival rates for PSC subjects with early-stage fibrosis were 100%, 100%, 100%, 92.31%, and 61.54%, compared with 70.37%, 64.51%, 52.78%, 42.22%, and 0% for those with advanced fibrosis, respectively. In contrast, transplant-free and hepatic decompensation-free survival did not differ between those with high or low spatial heterogeneity by LS variability or CoV.

After adjustment for multiple clinical variables, neither LS variability nor CoV was a predictor of transplant-free survival or hepatic decompensation-free survival, whereas LS remained a significant predictor of transplant-free survival (HR 1.52, 95% CI: 1.14–2.02, *p*=0.004) and hepatic decompensation (HR 1.94, 95% CI: 1.46–2.58, *p*<0.0001) (Table 3).

**TABLE 3:** Proportional hazard analyses of liver transplant or death and hepatic decompensation

	Liver transplant or death		Hepatic decompensation, liver transplant, or death			
	Univariate		Univariate		Multivariate	
	HR (95% CI)	*p*	HR (95% CI)	*p*	HR (95% CI)	*p*
Total bilirubin	1.00 (0.52–1.50)	0.63	1.00 (0.64–1.38)	0.75	—	—
Age	1.00 (0.94–1.05)	0.91	1.01 (0.96–1.05)	0.78	—	—
ALP	1.00 (1.00–1.00)	0.52	1.00 (1.00–1.00)	0.83	—	—
ALT	1.00 (1.00–1.01)	0.53	1.00 (1.00–1.01)	0.54	—	—
AST	1.00 (1.00–1.01)	0.94	1.00 (1.00–1.01)	0.86	—	—
Platelets	1.00 (1.00–1.00)	0.45	1.00 (1.00–1.00)	0.14	—	—
Creatinine	2.15 (0.17–27.8)	0.56	1.43 (0.16–13.14)	0.75	—	—
Albumin	1.00 (0.31–4.30)	0.83	0.94 (0.32–2.81)	0.91	**—**	—
INR	1.49 (0.13–17.41)	0.75	3.18 (0.30–34.01)	0.34	—	—
Sodium	1.18 (0.83–1.67)	0.36	1.05 (0.80–1.37)	0.72	—	—
LS, mean	*1.52* (*1.14–2.02*)	*0.004*	*1.94* (*1.46–2.58*)	<*0.0001*	*1.95* (*1.40–2.70*)	<*0.0001*
LS, CoV	1.00 (1.00–1.02)	0.70	1.00 (1.00–1.02)	0.71	—	—
LS, variability	1.03 (0.98–1.09)	0.22	1.06 (1.00–1.12)	0.06	1.00 (0.94–1.06)	0.98

Abbreviations: CoV, coefficient of variance; INR, international normalized ratio; LS, liver stiffness.

## DISCUSSION

Disease staging in PSC remains a challenge due to concerns of possible spatial heterogeneity of fibrosis within the liver, leading to under-staging or over-staging by liver biopsy or transient elastography. In the current study, we demonstrated that spatial heterogeneity of LS increases with advancing disease regardless of disease etiology, and importantly, had no impact on clinical outcomes. LS was the key driver of clinical outcomes in patients with PSC, independent of spatial heterogeneity.

A major advantage of MRE over histology or other measures of liver stiffness, such as transient elastography, for staging of disease, is the large area of the liver that is sampled with MRE. In several liver diseases, spatial heterogeneity of fibrosis has been documented on paired liver biopsies.^13^^,^^14^ More recently an analysis of reconstructed three-dimensional histological images of liver biopsies demonstrated that sampling error could result in over 20% of biopsies having a 1 stage difference by Scheuer staging and 2 stage difference by Ishak staging.^15^ Specifically, within PSC, this has been borne out in a randomized controlled trial of simtuzumab in which paired liver biopsies done 96 weeks apart in patients receiving placebo showed an almost random pattern of change with 22%, 33%, and 44% showing improvement, no change, or worsening by at least 1 Ishak stage, respectively.^16^

In MASLD, recent work by Kawamura et al.^11^ demonstrated that LS variability by MRE is associated with discordance between MRE-based staging and pathological staging. Because liver biopsy is rarely performed in PSC, a similar study has not yet been performed, and little is known about the spatial heterogeneity by MRE in PSC. In a proof-of-concept study that included 20 patients with PSC, CoV of LS by MRE was reported to be more heterogenous in patients with PSC compared with viral hepatitis, though the difference was small (21% vs. 18%, respectively).^10^ In contrast, we could not detect any difference in heterogeneity between patients with PSC compared with MASLD using the same measure of heterogeneity or within subgroups of patients with early or advanced fibrosis. These divergent findings may be due to differences in the comparator group (viral hepatitis vs. MASLD) or other unmeasured confounders. Our results are consistent with a prior study, which demonstrated that segmental LS is only weakly correlated with segmental biliary strictures^17^ and suggests that overall spatial heterogeneity is not different between PSC and MASLD.

Nevertheless, for historical reasons, liver biopsy remains the gold standard for staging in PSC and has been demonstrated to be a predictor of clinical events and survival.^4^^,^^16^^,^^18^ However, LS, which reflects not only fibrosis, but also cholestasis and inflammation, has also been shown to predict clinical outcomes in PSC.^5^^,^^6^^,^^19^ Our findings on the relationship between LS and prognostic models are also consistent with prior studies.^5^^,^^8^^,^^20^^,^^21^ Two prior studies demonstrated a similar correlation between LS and the Mayo Risk score.^10^^,^^21^ In addition, Tafur et al.^21^ reported that LS exhibited superior discriminatory ability compared with morphological intrahepatic stricture severity in distinguishing Mayo Risk Score-based risk groups. The above-mentioned study by Reiter and colleagues, which suggested greater heterogeneity in patients with PSC compared with viral hepatitis, also reported a marginally significant correlation between CoV and Mayo risk score (*r* = − 0.45; *p* = 0.05). However, our larger sample size did not show a correlation of CoV or variability with Mayo risk score or PREsTo score, and more importantly, neither measure of spatial heterogeneity was associated with the risk of clinical outcomes, including transplant/death or hepatic decompensation. Rather, LS alone was the driver of these clinical outcomes.

The amorphous nature of liver fibrosis in PSC and liver diseases at large is difficult to quantify. Current methods of staging liver fibrosis provide a single, discrete measurement meant to represent the entire organ without consideration of the heterogenous nature of fibrosis. Although we found no effect of the spatial heterogeneity of LS on clinical outcomes, more sophisticated measures, analyses, or understanding of the spatial nature and biology of liver fibrosis may identify patterns that are more likely to lead to disease progression and clinical outcomes. We employed 2 methods of measuring spatial heterogeneity from MRE data, which, like mean LS, provide a gross estimation of the variation of LS within a liver but lack specificity in terms of location or other features. Machine learning or other approaches, including deep learning models based on T2-weighted or post-contrast sequences,^22^^,^^23^ may find new patterns beyond current methods.

Despite the strengths of our study, including the use of 2 measures of LS spatial heterogeneity and detailed clinical data and outcomes, our study did have limitations. Our cohort was of a modest sample size, and validation studies with larger cohorts are anticipated. We also lacked histologic staging compared to our MRE-based staging. However, these limitations do not compromise the overall conclusions drawn from our study.

In conclusion, spatial heterogeneity of LS increases with progressive disease in both PSC and MASLD but is not associated with clinical outcomes in PSC. Mean LS predicts clinical outcomes in patients with PSC independent of LS spatial heterogeneity.
